# Effect of Electropolishing on the Microstructure and Tribological Properties of Electrolyte-Plasma Borided Layers on 30KhGSA Steel

**DOI:** 10.3390/ma18214867

**Published:** 2025-10-24

**Authors:** Laila Sulyubayeva, Nurbol Berdimuratov, Daryn Baizhan, Temirlan Alimbekuly, Balym Alibekova

**Affiliations:** 1 Research Center Surface Engineering and Tribology, Sarsen Amanzholov East Kazakhstan University, Ust-Kamenogorsk 070000, Kazakhstan; lsulyubayeva@gmail.com (L.S.); daryn.baizhan1@gmail.com (D.B.); temirlanalimbekuly@gmail.com (T.A.); balymalibekova304@gmail.com (B.A.); 2 Research School of Physical and Chemical Sciences, Shakarim University, Semey 071412, Kazakhstan

**Keywords:** electrolyte-plasma boriding, plasma-electrolytic polishing, boride layers, microstructure, wear, microhardness

## Abstract

The study investigates the effect of plasma-electrolytic polishing on the structure and wear resistance of 30KhGSA steel after plasma-electrolytic boriding. Plasma-electrolytic boriding was carried out in a boron-containing electrolyte at a temperature of 900 °C, which ensured the formation of a hardened modified layer consisting of a surface oxide layer, a subsequent zone composed of boride phases FeB and Fe_2_B, as well as a transitional martensitic zone. To remove brittle oxide phases and reduce surface roughness, plasma-electrolytic polishing in an alkaline solution was applied, which made it possible to form a smoother and more stable surface. The results showed that plasma-electrolytic boriding increases the microhardness up to 1500–1600 HV_0.1_, which is 5–6 times higher compared to untreated steel, and reduces the friction coefficient and wear rate. However, the borided layers exhibit brittleness and surface roughness. Subsequent plasma-electrolytic polishing made it possible to reduce surface roughness by nearly an order of magnitude, decrease the friction coefficient by more than 30%, and almost halve the wear rate. The obtained results confirm the high potential of this combined technology for strengthening structural steel components operating under high loads and severe wear conditions.

## 1. Introduction

Improving the service life of machine parts and tools through the diffusion saturation of the surface of metals and alloys with various chemical elements is one of the urgent tasks of modern materials science [1]. Chemical heat treatment (CHT) makes it possible to form a set of performance properties on the surface of products, which are either impossible to achieve through bulk alloying (nitriding, boriding) or economically impractical (chromizing, chromo-niobizing, etc.) [2]. At the same time, traditional methods of diffusion saturation, based on high-temperature isothermal holding with complete recrystallization of steel into the austenitic state, are accompanied by the overheating of the material, which leads to the deterioration of the structure and mechanical properties, except for hardness and wear resistance. In addition, such technologies are characterized by high energy consumption, cause warping of parts due to creep, and also lead to significant heat losses [3,4,5,6,7]. One of the ways to eliminate these drawbacks is the use of thermocycling modes, which make it possible to reduce the saturation temperature and shorten the process duration [8,9].

Boriding is one of the most effective CHT methods, providing the formation of hard boride phases, FeB and Fe_2_B, on the steel surface, which exhibit high microhardness (up to 2000 HV), enhanced wear and corrosion resistance, as well as stability at elevated temperatures [10]. Depending on the processing conditions, coatings with a thickness of 50–300 µm are formed, having either a single-phase (Fe_2_B) or a two-phase (FeB + Fe_2_B) structure [11]. When forming a two-phase layer (FeB/Fe_2_B), the FeB phase, which has a higher boron content, is characterized by brittleness and residual stresses at the interface with the Fe_2_B phase, which can lead to the formation of cracks and coating defects. At the same time, the single-phase structure based on Fe_2_B is considered more preferable, since it has high hardness, wear resistance, and stability due to lower thermal expansion coefficients compared to FeB [12]. Thus, the task of forming single-phase Fe_2_B layers with an optimal combination of hardness and ductility is a priority in the development of new boriding technologies [13]. In practice, various boriding methods are used: in powder mixtures (boron carbide, ferroboron, amorphous boron), in a gaseous medium (diborane, BCl_3_), in liquid media (electrolytic and electroless), and in the form of pastes and suspensions [14].

Traditional boriding processes, despite their effectiveness, are characterized by high energy consumption, significant duration (several hours at 800–1000 °C), and the formation of liquid and solid waste, which reduces their environmental and economic feasibility [15]. In this regard, new methods of intensifying diffusion saturation have been developed, aimed at reducing processing time and lowering energy consumption [16]. Among them, a special place is occupied by plasma-electrolytic boriding (PEB)—an innovative technology carried out in aqueous electrolytes under the action of micro-arc discharges [17].

During the PEB process, a high-voltage electric field (180–350 V) causes the formation of a vapor–gas envelope around the sample surface, inside which micro-arc discharges occur. These discharges generate localized heating of the surface up to 900–1100 °C, promoting the dissociation of boron-containing electrolyte components (e.g., Na_2_B_4_O_7_·10H_2_O) and intensive diffusion of atomic boron into the metallic substrate [18]. The process thus combines both electrochemical and thermal effects, providing accelerated boride layer growth at relatively low energy input. Depending on the polarity of the specimen, boriding can proceed in cathodic or anodic mode, enabling control over the microstructure and properties of the formed coatings.

For saturating the steel surface with boron during plasma-electrolytic boriding, the most commonly used boron-containing component is sodium tetraborate (borax) Na_2_B_4_O_7_·10H_2_O [19]. Upon melting, it loses crystallization water and dissociates with the formation of atomic boron, which participates in the diffusion saturation of the surface. However, the specific electrical conductivity of its aqueous solutions is not always sufficient for the effective course of the process [20]. In this regard, working electrolytes for cathodic PEB generally contain additional conductive additives, such as sodium carbonate (Na_2_CO_3_) and sodium hydroxide (NaOH). These components increase the electrical conductivity of the solution, help stabilize the plasma-discharge mode, and ensure uniform saturation of the surface with boron. Additional salts may also influence the coating morphology and its phase composition [21].

Thus, PEB is a promising direction in the development of surface hardening technologies for steels [22]. It makes it possible to significantly reduce processing time, lower energy consumption, ensure environmental safety, and form coatings with an improved set of performance properties, which makes this method a competitive alternative to traditional types of boriding [23]. At the final stage of the plasma-electrolytic boriding process, interaction between the active electrolyte particles and the heated surface may lead to the formation of thin oxide layers (mainly Fe_2_O_3_ and Fe_3_O_4_) [24]. The thickness of this oxide film depends on the electrolyte composition, temperature, and electrode polarity. On the one hand, this layer can enhance corrosion resistance; on the other hand, its excessive growth reduces coating adhesion and promotes cracking. To eliminate these drawbacks, plasma-electrolytic polishing (PEP) can be applied as a post-treatment step. PEP operates under the anodic plasma regime (typically 250–350 V), where a thin vapor–gas envelope forms around the anode surface. Within this region, plasma microdischarges and anodic dissolution occur simultaneously, resulting in selective removal of oxide films, smoothing of micro-roughness, and reduction in surface defects [25]. This process significantly improves the microgeometry and functional properties of the treated surface while maintaining the integrity of the underlying diffusion layer. As a result, the sequential combination of PEB and subsequent PEP allows the formation of dense, homogeneous, and smooth boride layers with optimized tribological characteristics and enhanced operational performance [26].

Thus, taking into account the analysis of traditional boriding methods and their limitations, the aim of this work is a comprehensive study of the plasma-electrolytic boriding process of structural steel 30KhGSA and subsequent plasma-electrolytic polishing for the formation of multilayer hardened layers on the material surface.

## 2. Materials and Methods

As the material for the study, structural alloy steel 30KhGSA was used, which is widely applied in the production of parts operating under conditions of intensive friction and wear, such as bushings, gears, and bearing rings. Samples with dimensions of 2 × 2 × 2 cm^3^ were cut from 30KhGSA steel bars [27]. Before the experiment, the surface of the samples was subjected to sequential mechanical preparation: grinding with abrasive paper of grit size from P100 to P2000, followed by polishing with diamond paste with a particle size of 0.25–0.5 µm, and then cleaning in ethyl alcohol. The chemical composition of 30KhGSA steel (according to GOST 4543–71) [28] is given in Table 1.

**Figure 1:**
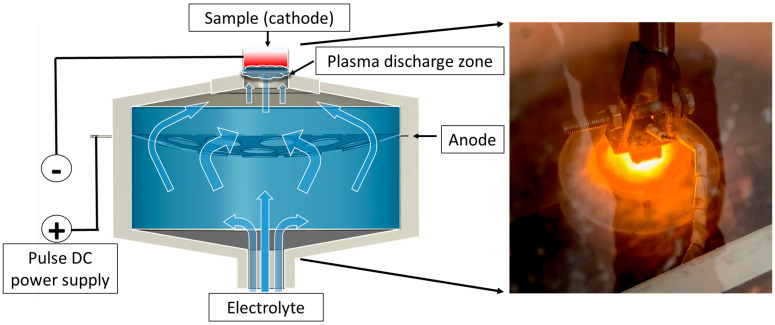
Schematic diagram of the plasma-electrolytic boriding process of the samples.

The plasma-electrolytic boriding process was carried out on a specially designed setup (Figure 1). Samples made of 30KhGSA steel were fixed on a holder and connected as the cathode. A stainless-steel disk with a system of holes ensuring uniform electrolyte distribution was used as the anode. The electrolyte was supplied through a conical nozzle made of insulating material; the flow passed through the anode in the form of a concave disk and was directed onto the cathode surface (the sample being treated) through the upper outlet of the cap. Voltage to the anode and cathode was supplied from a direct current (DC) power source. When the specified electric field parameters were reached, micro-arc discharges occurred on the sample surface, providing localized heating and accelerated diffusion of boron atoms. An aqueous solution with boron-containing additives was used as the working electrolyte, and its composition is presented in Table 2. At the initial stage of the experiment, the steel samples were immersed in the electrolyte flow at a rate of 1.2 L/min, with the distance between the cathode and the anode being 4 mm. Plasma-electrolytic boriding was carried out at a voltage of 170 V for 20 min. The temperature of the samples was monitored using a thermocouple inserted into a pre-drilled hole in the sample body, which ensured accurate temperature measurement during processing. During the experiment, the surface temperature was maintained at 900 °C, which promoted the formation of a diffusion boride layer on the surface.

**Figure 2:**
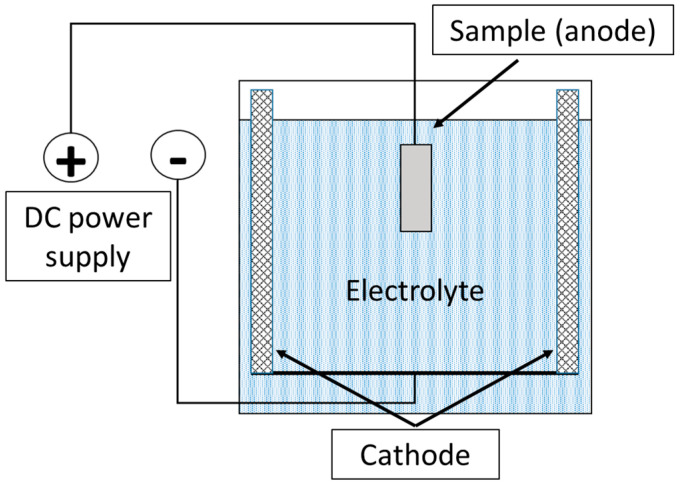
Schematic diagram of the plasma-electrolytic polishing process of the samples.

To remove oxide layers after plasma-electrolytic boriding, a finishing treatment method—plasma-electrolytic polishing (PEP)—was developed. This method is based on the action of pulsed electrical discharges occurring in the vapor–plasma envelope (VPE) formed around the anodic sample immersed in the electrolyte. The workpiece was connected as the anode, to which positive voltage was applied, while the cathode was the body of the working bath, equipped with lead plates evenly arranged along its perimeter (Figure 2). The electrolyte, the composition of which is given in Table 2, was used as the working solution. The process was carried out at a current density of 0.5 A/cm^2^, while the surface temperature of the treated samples did not exceed 60 °C. The treatment duration was 3 min.

**Table 2:** Composition of electrolytes used in plasma-electrolytic boriding and plasma-electrolytic polishing.

Process	Component	Formula	Purpose	Concentration
Plasma-electrolytic boriding	Sodium tetraborate (borax)	Na_2_B_4_O_7_·10H_2_O	Boron source	30 wt.%
	Sodium carbonate	Na_2_CO_3_	Increase in electrical conductivity	10 wt.%
	Deionized water	H_2_O	Solvent	60 wt.%
Plasma-electrolytic polishing	Sodium hydroxide	NaOH	Removal of oxides (Fe_2_O_3_, Fe_3_O_4_), anodic dissolution	6 wt.%
	Sodium silicate (opt.)	Na_2_SiO_3_	Stabilization of the PPT	4 wt.%
	Sodium phosphate (opt.)	Na_3_PO_4_	Buffering/complex formation	2 wt.%
	Deionized water	H_2_O	Solvent	88 wt.%

The phase composition of the studied samples was determined using an X’PertPro X-ray diffractometer (PANalytical B.V., Almelo, The Netherlands) with CuKα radiation at 40 kV and 30 mA. The measurements were carried out in the angular range of 20° < 2θ < 90° with a step size of 0.02°, and an exposure time of 5 s. The processing of diffractograms and quantitative phase analysis were performed using PowderCell 2.4 software. The identification of reflections was performed using standard reference cards: α-Fe—PDF 00-006-0696, Fe_2_B—PDF 00-036-1332, FeB—PDF 01-076-0092, Fe_2_O_3_—PDF 00-033-0664, and Fe_3_O_4_—PDF 00-019-0629. The microstructure of the samples was revealed by chemical etching in a 4% nitric acid (HNO_3_) solution in ethyl alcohol. Optical metallographic studies were carried out using an Olympus microscope (Olympus Corporation, Tokyo, Japan) in reflected light under bright-field mode. Additionally, microstructural analysis was performed on cross-sections of borided samples prepared according to the standard metallographic procedure (grinding, polishing, and etching), using a SEM3200 scanning electron microscope (CIQTEK Co., Ltd., Hefei, China). The elemental composition of the modified surface layers was analyzed using energy-dispersive X-ray spectroscopy (EDS) with an Oxford Instruments (UK) detector (Oxford Instruments, Abingdon, United Kingdom) integrated into the scanning electron microscope. The microhardness of the boride layers was measured in accordance with DIN EN ISO 14577-1 [29] and ASTM E2546 standards [30] using a FISCHERSCOPE^®^ HM2000 S microhardness tester (Helmut Fischer GmbH, Sindelfingen, Germany) equipped with Vickers diamond indenters. The applied load was 0.98 N with a dwell time of 10 s. The measurements were carried out on the cross-section of the samples, from the surface of the boride layer toward the metallic matrix. The hardness profile was plotted based on the measurement results taken at points evenly distributed across the layer thickness. To improve the reliability of the results, three independent measurements were taken at each point in different areas, after which the average microhardness value was calculated. Tribological tests were performed on a TRB3 tribometer (Anton Paar, Austria) using the “ball-on-disk” configuration in accordance with the ASTM G99 standard [31]. A Si_3_N_4_ ball with a diameter of 6 mm was used as the counterbody. The tests were conducted under a normal load of 6 N, at a sliding speed of 2 cm/s (0.02 m/s), with a wear track radius of 2 mm and a total sliding distance of 60 m. All experiments were carried out under dry sliding conditions at room temperature (24 ± 5 °C) and relative humidity of 12 ± 5%. Each test was repeated three times for every experimental condition to ensure reproducibility and statistical reliability of the results obtained. The average values and standard deviations were calculated based on the three measurements. After testing, the wear track depth and cross-sectional area were analyzed using a Taylor Hobson surface profilometer (UK) in accordance with the ISO 4287 standard [32]. The detailed tribological test parameters are summarized in Table 3.

## 3. Results and Discussion

**Figure 3:**
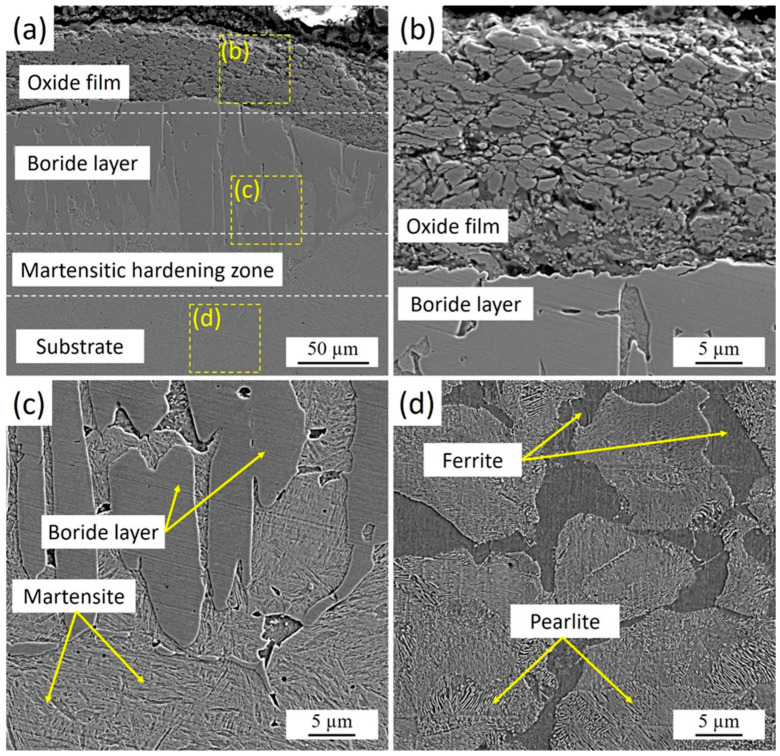
Microstructure of the cross-section of 30KhGSA steel samples after plasma-electrolytic boriding: (**a**) general view of the structure with an oxide film, boride layer, and martensitic hardening zone; (**b**) enlarged fragment of the oxide film; (**c**) structure of the boride layer and martensite; (**d**) ferrite–pearlite structure of the substrate.

Figure 3 presents the cross-sectional microstructure of 30KhGSA steel after plasma-electrolytic boriding. A thin oxide film is observed on the specimen surface, formed at the final stage of the process as a result of the interaction between active electrolyte components and the heated metal surface. Beneath the oxide layer lies a dense boride zone characterized by a needle-like crystal morphology, which is typical of the FeB and Fe_2_B phases. The kinetics of boride layer formation is strongly influenced by the chemical composition and structural state of the steel substrate. For pure iron, unalloyed low-carbon, and low-alloy steels, the boride layer usually develops with a sawtooth-shaped “boride/substrate” interface, caused by intensive boron diffusion and anisotropic growth of boride phase crystals. With increasing carbon content and/or alloying elements, the serrated morphology of the interface tends to smooth out due to the thermodynamic stabilization of the matrix and a reduction in diffusion mobility. In high-alloy steels, the presence of alloying elements promotes the formation of a relatively flat interface, reflecting a more uniform boride growth and a lower diffusion gradient [33].

Below the borided layer, a martensitic hardening zone was identified. This zone forms as a result of rapid surface cooling immediately after plasma-electrolytic boriding. During processing, micro-arc discharges locally heat the surface to high temperatures, while the surrounding electrolyte simultaneously acts as an effective quenching medium. The direct contact between the hot surface and the relatively cold electrolyte ensures an extremely high cooling rate (on the order of 10^3^–10^4^ K/s), leading to the transformation of austenite into martensite. This mechanism produces a hardened transition layer beneath the boride zone, creating a smooth gradient in mechanical properties from the hard FeB/Fe_2_B surface layer to the more ductile substrate.

**Figure 4:**
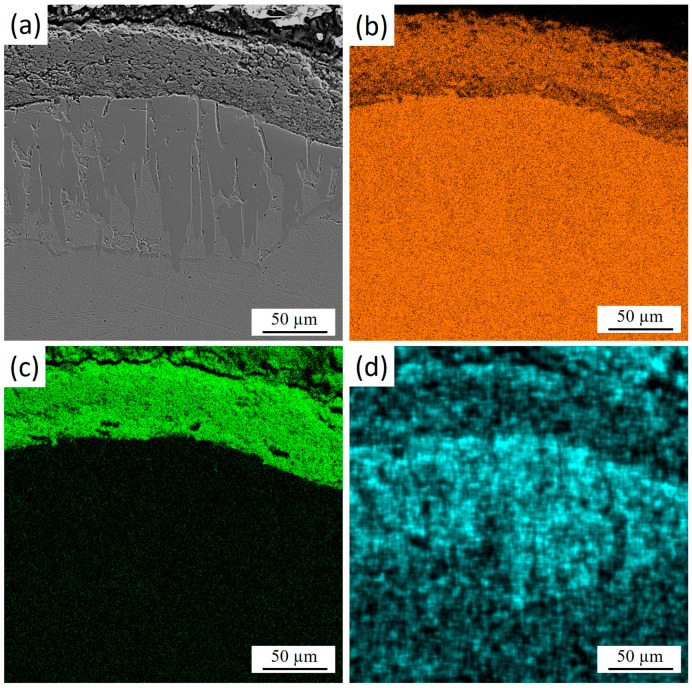
Cross-section of 30KhGSA steel after plasma-electrolytic boriding: (**a**) microstructure of the layer; EDS elemental mapping—(**b**) distribution of Fe, (**c**) distribution of O, (**d**) distribution of B.

Figure 4 shows the cross-section of 30KhGSA steel after plasma-electrolytic boriding with the results of energy-dispersive analysis confirming the formation of a gradiently inhomogeneous modified layer. Elemental mapping indicates a regular redistribution. Iron (Figure 4b) maintains a high concentration in the bulk of the substrate, while its content decreases in the near-surface zone due to substitution by boron and oxygen. Oxygen (Figure 4c) is concentrated mainly in the upper part of the coating, which indicates the formation of oxide phases. Boron (Figure 4d) shows local accumulation in the near-surface region, which confirms the formation of boride compounds. The overall results demonstrate the formation of a multicomponent boride–oxide layer with a clearly pronounced gradient zonality in elemental composition.

**Figure 5:**
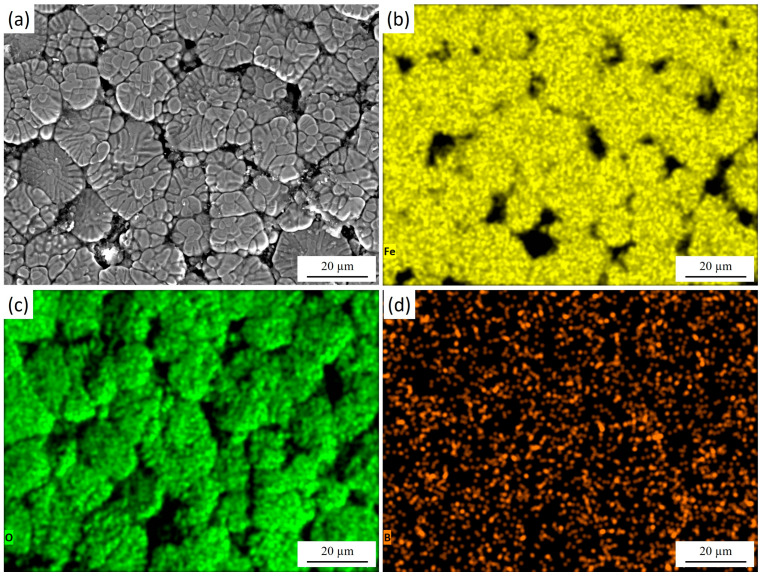
Surface morphology of 30KhGSA steel after plasma-electrolytic boriding: (**a**) surface microstructure; EDS elemental mapping—(**b**) distribution of Fe, (**c**) distribution of O, (**d**) distribution of B. The quantitative EDS analysis shows the following elemental composition (at.%): Fe—51.25, O—44.89, B—3.86.

Figure 5 presents the surface morphology of 30KhGSA steel after plasma-electrolytic boriding together with the results of EDS elemental mapping. The microstructural image (Figure 5a) reveals a characteristic granular surface with pronounced grain boundaries and a heterogeneous relief, which is associated with intensive diffusion saturation during the treatment. The elemental mapping confirms the chemical composition of the modified layer. During plasma-electrolytic boriding, the specimen acted as the cathode, and boron-containing species from the electrolyte were activated in the discharge channels and transported toward the surface. This elemental distribution results from the combined effect of cathodic plasma discharges and subsequent cooling in the electrolyte. Boron (Figure 5d) exhibits a dispersed distribution across the entire surface, confirming its successful incorporation into the diffusion layer and the formation of boride phases (FeB, Fe_2_B). Iron (Figure 5b) is relatively uniformly distributed in the bulk, while its concentration decreases along the grain boundaries. Oxygen (Figure 5c) is mainly concentrated on the surface and in the near-surface regions, indicating the formation of oxide phases. After completion of the process, rapid cooling of the specimen in the electrolyte leads to oxidation of the still-hot surface. As a result, a thin oxide film forms on top of the boron-enriched layer, leading to the coexistence of oxide (Fe_2_O_3_, FeO) and boride (FeB, Fe_2_B) phases within the modified surface structure.

**Figure 6:**
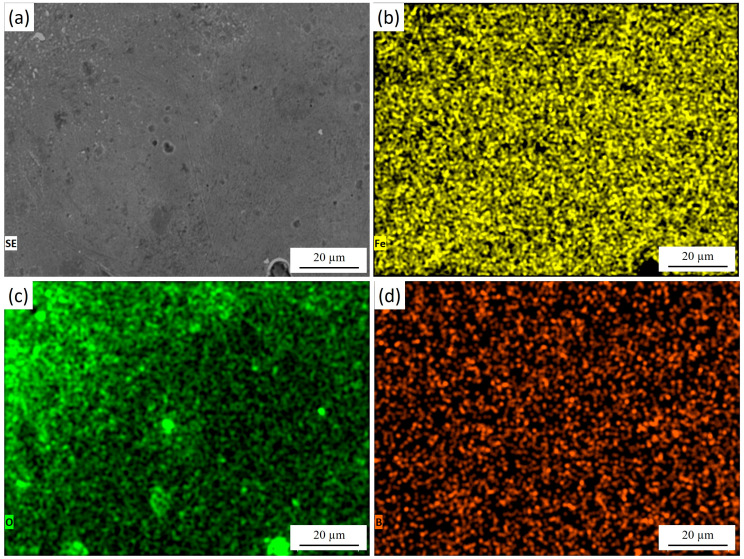
Surface of 30KhGSA steel after plasma-electrolytic polishing: (**a**) surface microstructure; EDS elemental mapping—(**b**) distribution of Fe, (**c**) distribution of O, (**d**) distribution of B. The quantitative EDS analysis shows the following elemental composition (at.%): Fe—83.88, O—8.04, B—8.09.

Figure 6 presents the surface morphology of 30KhGSA steel after plasma-electrolytic polishing together with the results of EDS elemental mapping. The microstructural image (Figure 6a) shows a smooth and uniform surface without a pronounced granular relief, indicating the effective removal of micro-roughness and surface reaction products. The elemental maps confirm the chemical composition of the modified layer. Iron (Figure 6b) is evenly distributed across the entire surface, reflecting the preservation of the metallic substrate after polishing. Oxygen (Figure 6c) is mainly detected in the near-surface region, suggesting that after polishing, the interaction with the electrolyte promotes the formation of a very thin and uniform oxide film. Boron (Figure 6d) is dispersed across the surface, confirming the retention of boride inclusions after the polishing treatment. During plasma-electrolytic polishing, the previously formed thick oxide layer is effectively removed, exposing a clean and dense boride surface. However, upon completion of the process and during cooling in the electrolyte, a thin protective oxide film naturally reforms on the surface—a phenomenon typical for metallic materials. As a result, the final surface consists of a smooth boride layer covered by a nanometer-scale oxide film that provides additional passivation and stability.

**Figure 7:**
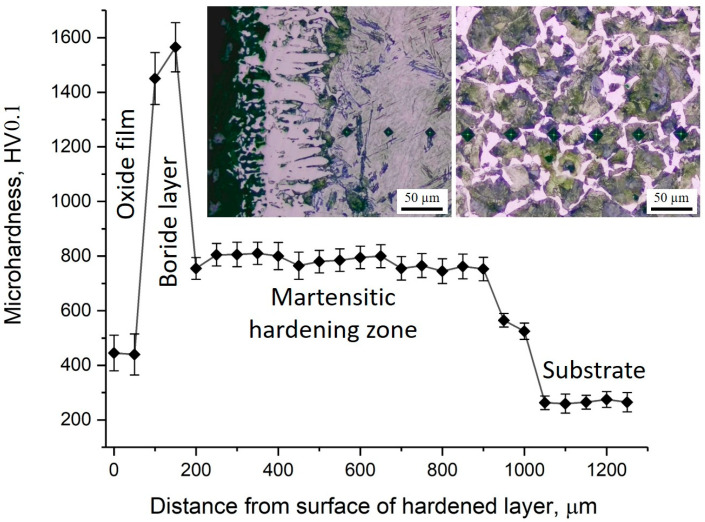
Microhardness distribution across the cross-section of 30KhGSA steel after electrolyte-plasma boriding with corresponding microstructural images of different zones of the hardened layer.

The distribution of microhardness across the depth of the borided layer of 30KhGSA steel and the corresponding microstructural images are shown in Figure 7. On the sample surface, a thin oxide layer with relatively low hardness (about 400–500 HV0.1) is observed, beneath which a boride layer is formed with maximum microhardness values reaching 1500–1600 HV0.1. This layer provides the main contribution to the improvement of the wear resistance of the treated surface. Below lies the martensitic hardening zone with a microhardness of about 700–850 HV0.1, which serves as a transition layer between the hard boride coating and the more ductile substrate. Starting from a depth of about 900–1000 µm, a gradual decrease in microhardness is observed down to the level of the initial substrate (~250–350 HV0.1), represented by the ferrite–pearlite structure. In the microstructural images, the boride crystals with needle-like morphology, the martensitic hardening zone, and the structure of the initial substrate are clearly distinguishable. Thus, the obtained data confirm the multilayer structure of the modified surface, where the oxide film, boride layer, and martensitic zone provide high hardness and wear resistance, while the core retains the ductility and toughness characteristic of 30KhGSA steel.

**Figure 8:**
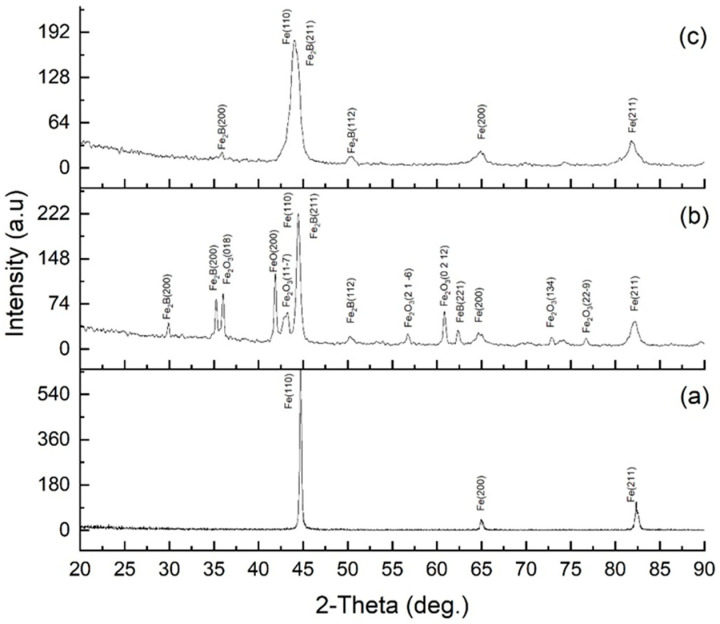
XRD patterns of 30KhGSA steel: (**a**) initial state; (**b**) after electrolyte-plasma boriding; (**c**) after plasma-electrolytic polishing.

X-ray diffraction analysis (Figure 8) made it possible to determine the evolution of the phase composition of 30KhGSA steel in the initial state, after plasma-electrolytic boriding, and subsequent plasma-electrolytic polishing. For the initial steel (a), the diffraction peaks of α-Fe are observed at 2θ ≈ 44.7° (Fe(110)), 65° (Fe(200)), and 82° (Fe(211)), which corresponds to a ferrite–pearlite structure without additional phases. After plasma-electrolytic boriding (b), intense peaks of boride phases Fe_2_B and FeB (Fe_2_B(200), Fe_2_B(211), FeB(112), etc.) are recorded, as well as peaks corresponding to iron oxide compounds (FeO, Fe_2_O_3_), indicating the formation of a multilayer coating that includes borides and a surface oxide film. The formation of FeO and Fe_2_O_3_ oxide phases is caused by high-temperature surface oxidation, which is a characteristic feature of plasma-electrolytic processes [34]. Subsequent plasma-electrolytic polishing led to the removal of oxide-related peaks, while Fe_2_B peaks remained dominant and FeB peaks weakened, indicating densification and stabilization of the boride layer. The noticeable broadening of α-Fe peaks after both treatments is attributed to microstructural refinement, increased defect density, and lattice strain in the near-surface region. During boriding, local heating and rapid quenching (10^3^–10^4^ K·s^−1^) induced a fine-lath martensitic structure beneath the boride layer, accompanied by high dislocation density, microstresses, and subgrain formation, which caused FWHM broadening due to crystallite size reduction and strain effects. Boron diffusion and compositional gradients further contributed to microstrain-induced broadening. After polishing, despite partial stress relaxation, the subsurface fine-grained martensitic and partly tempered structure with residual lattice distortions remained, maintaining the broadened α-Fe peaks relative to the initial state. This indicates the stabilization of a defect-rich, nanostructured subsurface region formed during boriding, responsible for the improved hardness and mechanical performance of the modified layer.

**Figure 9:**
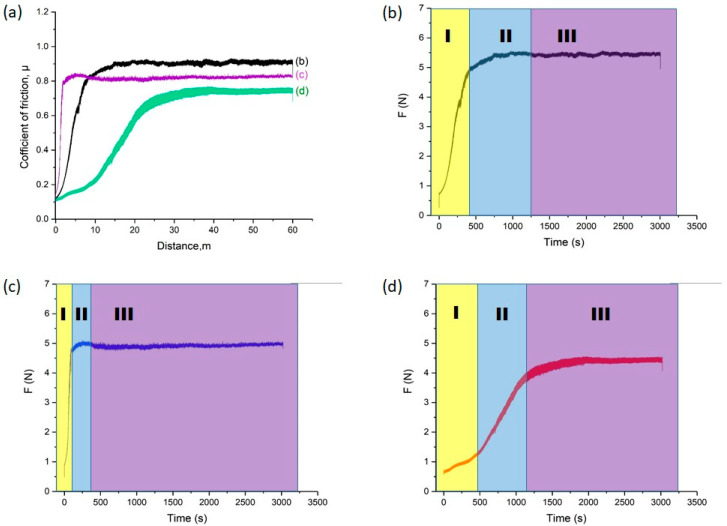
Results of tribological tests: (**a**) friction coefficient of 30KhGSA steel samples in the initial state (**b**), after boriding (**c**), and after subsequent plasma-electrolytic polishing (**d**); friction force versus time with indication of friction stages (I—running-in, II—formation of the transition layer, III—steady-state friction).

Figure 9 shows the results of tribological tests of the studied samples: friction coefficient as a function of sliding distance (a) and friction force versus time for the initial 30KhGSA steel (b), after plasma-electrolytic boriding (c), and after subsequent plasma-electrolytic polishing (d). For the initial steel (Figure 6b), three characteristic regions are observed: at the running-in stage (I), a rapid increase in the friction coefficient occurs due to the establishment of contact and the transition to plastic interaction; in region II, an oxide film (glaze layer) is formed, stabilizing the friction process; in region III, steady-state friction with relatively stable coefficient values is recorded, indicating the dominance of the adhesive wear mechanism. After boriding (Figure 9c), the curve behavior changes: in region I, a sharp increase in friction force is observed, associated with the rough and brittle surface of the boride layer prone to chipping and the formation of abrasive particles; in region II, the abrasive wear mechanism dominates due to the action of large and fine particles; in region III, friction stabilizes but remains higher compared to the initial steel, which is related to the presence of hard and brittle oxide phases (FeO, Fe_2_O_3_) and the FeB boride phase. After polishing (Figure 9d), the surface becomes smoother and less prone to chipping: in region I, a gradual increase in the friction coefficient is recorded; in region II, a progressive establishment of a stable process occurs; and in region III, steady friction is observed at a level lower than that of the borided layer but higher than that of the initial steel, which is explained by surface smoothing while retaining the hard boride layer. Thus, the conducted studies show that boriding significantly alters the friction mechanisms, shifting them from adhesive to abrasive, while subsequent plasma-electrolytic polishing contributes to reducing the friction coefficient and stabilizing the wear process.

**Figure 10:**
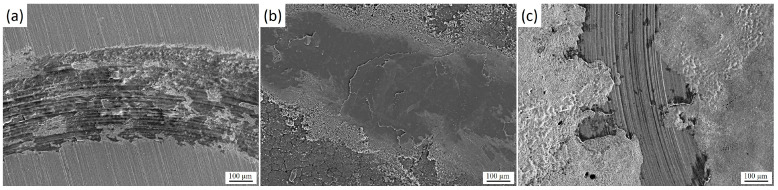
Morphology of wear tracks of 30KhGSA steel after tribological tests: (**a**) initial state, (**b**) after plasma-electrolytic boriding, (**c**) after subsequent plasma-electrolytic polishing.

Figure 10 shows the morphology of the wear tracks after tribological tests: initial 30KhGSA steel (a), after plasma-electrolytic boriding (b), and after subsequent plasma-electrolytic polishing (c). For the initial sample (Figure 10a), the formation of an oxide layer and the so-called glaze layer is characteristic, arising from local temperature increase in the friction zone. Signs of delamination of this layer are observed, along with the presence of fine wear products. In the case of the borided sample (Figure 10b), pronounced plowing grooves, wear debris, as well as elements of abrasive and delamination wear mechanisms are recorded, which is associated with the brittleness of the formed FeO and Fe_2_O_3_ phases and the tendency of the layer to chipping [35,36]. For the polished samples (Figure 10c), a significantly smoother surface is observed with a limited amount of wear debris and weakly expressed grooves, which indicates a reduction in wear intensity and the predominance of a more stable abrasive wear mechanism.

The results presented in Table 4 clearly illustrate the identified trends. For the initial 30KhGSA steel, the friction coefficient was 0.840, the wear rate reached 2.276 × 10^−4^ mm^3^/N·m, and the surface roughness did not exceed 1.2 µm. After plasma-electrolytic boriding, the friction coefficient decreased to 0.806, and the wear rate was reduced by more than half (1.036 × 10^−4^ mm^3^/N·m), which is attributed to the formation of a hard boride layer composed mainly of FeB and Fe_2_B phases. However, the surface roughness increased to 2.52 ± 0.25 µm as a result of the brittle nature of the FeB phase and the occurrence of microchipping and surface irregularities caused by the diffusion growth mechanism [37,38].

The most favorable tribological characteristics were obtained after plasma-electrolytic polishing: the friction coefficient decreased markedly to 0.571, the wear rate slightly decreased to 9.235 × 10^−5^ mm^3^/N·m, and the surface roughness significantly decreased to 0.3 ± 0.35 µm. This indicates that polishing effectively smooths the surface, removes oxide residues and brittle FeB fragments, and reduces micro-asperities, thereby minimizing the real contact area and frictional adhesion.

The relatively small decrease in the wear rate compared with the pronounced reduction in the friction coefficient and roughness can be explained by the fact that plasma-electrolytic polishing mainly modifies the surface topography and frictional behavior, while the subsurface hardness and boride layer thickness remain nearly unchanged. Thus, the resistance to material removal during wear is still governed by the intrinsic hardness and integrity of the borided diffusion layer.

Overall, the combined analysis of the wear track morphology, microstructure, and quantitative data confirms that plasma-electrolytic boriding substantially increases surface hardness and wear resistance, albeit at the cost of increased roughness and brittleness. In contrast, the subsequent plasma-electrolytic polishing significantly improves the tribological stability by reducing the friction coefficient and surface roughness, while maintaining the high wear resistance of the borided layer.

**Table 4:** Tribological characteristics and surface roughness of 30KhGSA steel in the initial state, after plasma-electrolytic boriding, and subsequent plasma-electrolytic polishing.

Sample	Friction Coefficient	Wear Rate (mm^3^/N·m)	Surface Roughness, Ra (µm)
Initial 30KhGSA steel	0.840 ± 0.020	(2.276 ± 0.11) × 10^−4^	≤1.2
After plasma-electrolytic boriding	0.806 ± 0.018	(1.036 ± 0.08) × 10^−4^	2.52 ± 0.25
After subsequent plasma-electrolytic polishing	0.571 ± 0.015	(0.923 ± 0.07) × 10^−4^	0.300 ± 0.035

## 4. Conclusions

The conducted studies showed that plasma-electrolytic boriding of 30KhGSA steel provides the formation of a multilayer hardened structure, including a surface oxide layer, FeB and Fe_2_B boride phases with needle-like morphology, as well as a transitional martensitic zone, which together lead to a significant increase in microhardness (up to 1600 HV_0.1_) and wear resistance compared to the initial steel. At the same time, the borided layers are characterized by increased brittleness and higher roughness (up to 2.5 µm), which promotes the development of abrasive and delamination wear mechanisms and is accompanied by an unstable friction coefficient. The introduction of subsequent plasma-electrolytic polishing made it possible to eliminate brittle protrusions and surface defects, significantly reduce roughness (down to 0.3 µm), decrease the friction coefficient by more than 30%, and reduce the wear rate by almost half. In addition, this process ensured the removal of oxide phases (FeO, Fe_2_O_3_), stabilization of the boride layer structure, and improvement of its service performance. The obtained results allow us to conclude that the combined technology, including plasma-electrolytic boriding and subsequent plasma-electrolytic polishing, provides an optimal combination of high hardness, wear resistance, and surface quality, and therefore can be recommended for the modification of structural steels and strengthening of components operating under conditions of intensive friction, high contact loads, and aggressive environments.

## Figures and Tables

**Table 1:** Chemical composition of 30KhGSA steel, wt.%.

C	Si	Mn	Ni	S	P	Cr	Cu
0.28–0.34	0.9–1.2	0.8–1.1	up to 0.3	up to 0.025	up to 0.025	0.8–1.1	up to 0.3

**Table 3:** Tribological test parameters according to ASTM G99 standard (“ball-on-disk” configuration).

Tribological Experimental Test Conditions (ASTM G99)	Parameter
Normal force (N)	6
Sliding speed (m/s)	0.02
Sliding distance (m)	60
Ball diameter (mm)	6 (Si_3_N_4_ ball)
Environment	Dry (ambient air)
Track diameter (mm)	4
Temperature (°C)	24 ± 5
Relative humidity (RH, %)	12 ± 5
Number of repetitions	3

## Data Availability

The original contributions presented in this study are included in the article. Further inquiries can be directed to the corresponding author.
